# Editorial: Surveillance of language development in pre-school children

**DOI:** 10.3389/fped.2022.1042094

**Published:** 2022-11-17

**Authors:** Daniel Holzinger, David Saldaña, Johannes Fellinger

**Affiliations:** ^1^ Research Institute for Developmental Medicine, Johannes Kepler University of Linz, Linz, Austria; ^2^ Institute of Neurology of Senses and Language, Hospital of St. John of God, Linz, Austria; ^3^ Institute of Linguistics, Faculty of Humanities, University of Graz, Graz, Austria; ^4^ Department of Developmental and Educational Psychology, Universidad de Sevilla, Seville, Spain; ^5^ Division of Social Psychiatry, University Clinic for Psychiatry and Psychotherapy, Medical University of Vienna, Vienna, Austria

**Keywords:** language surveillance, language screening, preschool, public health, primary pediatric care

**Editorial on the Research Topic** Surveillance of language development in Pre-school children

Language disorders are among the most frequent developmental disorders and can have a profound impact on academic and vocational development, mental health, and quality of life from childhood into adolescence and adulthood. Since there is increasing evidence for the effectiveness of intervention, particularly if offered timely and in a family-centered way, early identification of significant language delays is crucial in preventive health care. Due to the high variance of language trajectories in the early years, continuous monitoring of language development rather than single-point screening is indicated.

The collection of articles on this research topic that includes mainly empirical research, as well as a systematic review and meta-analysis by So & To, is an innovative contribution to the field of high relevance for clinical practice focusing on the type of administration (proxy vs. direct), language skills or clinical markers, the consideration of environmental factors (including special populations), the concurrent or predictive character of the screenings, and the feasibility of systematic language surveillance in total populations.

## Administration of proxy or direct screening

The findings of studies including parent report screenings support the conclusion of the systematic review by So & To, who show a comparable level of accuracy of parent reports as compared to screenings administered by trained examiners. For children at the age of two years (Holzinger et al.) and three to four years (Doove et al., Holzinger et al., Dockrell et al.) parental screenings achieved a high accuracy. Holzinger et al. demonstrated, for two-year screening, that the parent report as stage 1 (followed by a stage 2 direct evaluation by the pediatrician limited to those failing at stage 1) resulted in good predictive validity of language delay, even one year after screening administration. This finding points to the effectiveness of screening instruments based on a combination of direct child assessment and proxy reports, in line with the recently reported results for a Dutch well child language screening protocol (1).

## Screenings based on language ability

Various studies suggest greater precision of instruments based on the child's language ability, compared to those based on clinical markers such as non-word repetition or sentence repetition. This could be due to the high variability found in non-word and sentence repetition in children with language disorder (2). The studies included in the current research topic are mainly language-based and result in good or even excellent accuracy (Holzinger et al., Holzinger et al., Holzinger et al., Holzinger et al., Dockrell et al., Doove et al.). In addition to the assessment of child language skills, parental concerns about language development are found to be predictive of language development trajectories as shown by Holzinger et al. and Doove et al. for language development from age 2–3 and 3–4 years, respectively. Lüke et al's contribution shows for a sample of bilingual infants that the absence of the prelinguistic skill of index finger pointing at the age of 12 months, which shows intentional communication and the ability to initiate joint attention, seems to be an early indicator of language delay at the age of 2 years, in line with findings for monolingual populations (3). In conclusion, the evaluation of language abilities and proximal precursors of linguistic skills, direct or indirect, and parental concerns about their child's language development should be considered key components of effective language screening instruments.

## Environmental factors

Language is the product of a complex interplay of biological and environmental factors over time. Factors related to a child's home environment can either buffer or increase biological risk and help to understand children's developmental pathways and the early identification of risk. Eadie et al. demonstrate the potential use of early cumulative risk factors related to the home learning environment (e.g., number of books in the home, frequency of reading, and maternal education, maternal language, and mental health), including parent-child interaction (in addition to characteristics of the child) in the prediction of low language outcomes at 7 years. Many of these factors could probably be included in developmental surveillance programs, although the feasibility of such a recommendation remains to be demonstrated.

## Concurrent or predictive screenings

The highly dynamic nature of language development trajectories usually results in a non-satisfying rate of children with later language difficulties missed by a screening at an earlier point of time and/or in screening-fails who turn out to achieve an average language level later-on without having undergone any specific intervention (false positives). As expected, screening tools with longer screening diagnostic intervals demonstrate lower sensitivity than those using short intervals, as shown by the systematic review included in this research topic (So & To). Current findings demonstrate that, because a significant number of children with negative screening results at an earlier point of time develop language difficulties later continuous monitoring of language development (re-screening) and the capturing of environmental effects on language development are required. To avoid early over-identification associated with unnecessary irritation of parents, cost of follow-up investigation and/or interventions with high positive predictive values of the screenings are highly relevant for a population-based implementation of a screening tool. Holzinger et al. demonstrated good predictive validity of a two-stage screener that included a parent report of expressive vocabulary and two-word combinations, parent concerns about language development, and pediatric assessment of word comprehension. In summary, including language comprehension and parental concerns about language development increases the predictive quality of language screenings.

## Feasibility

As pointed out in the literature (4), evidence of feasibility of screening measures in regular preventive medical care settings is insufficient. However, the proof of feasibility is essential for the introduction of universal language screening. The studies of our working group that resulted in accurate screening measures for use in pediatric primary care (at the age of 2 and 3 years;  Holzinger et al. and Holzinger et al.) and pre-school settings (age of about 4 ½ years; Holzinger et al. and Holzinger et al.) were all implemented with large populations of non-selected children and within the regular system of preventive health care. Acceptability by screeners, parents, and children was rated as high in accordance with high rates of completed screening procedures. It should be noted that even the integration of screening within the time constraints of regular pediatric care was mainly estimated to be well possible. The combination of parent reports and—possibly as a second stage—assessments by trained screeners can contribute to an efficient administration in regular preventive health care. However, in their study on language screenings in disadvantaged populations including a significant number of non-English speaking parents, Dockrell et al. reported low completion rates of parent questionnaires.

## Special populations

Dockrell et al. confirmed higher rates of language difficulties in socially disadvantaged populations and showed that a shortened version of a parent questionnaire on their child's language performance was an effective measure that captured the language learning needs of children before they enter nursery schools. The low rate of returned screening forms (38.6%) points to the remaining challenges involved in the use of parent reports with this population.

For children in their penultimate year of kindergarten who grow up multilingually with a minority language as their dominant language, Holzinger et al. demonstrated that a screening targeting expressive grammatical skills in the majority language with bilingual norms achieves high accuracy in the identification of children with language disorders.

## Screening for increased risk for deficits in language-related skills

Schöfl et al. present a promising app-based screening tool for universal use at school entry to predict word-reading difficulties through a combination of phonological information processing and linguistic skills. In two small groups of Arabic speaking children and adults who stutter, significant correlations between non word repetition skills and the percentage of stuttered syllables indicate that nonword repetition tasks might be useful for the early identification of stuttering (Alsulaiman et al.).

## Conclusions and future directions

This research topic demonstrates the availability of accurate and feasible tools for use in developmental surveillance programs to identify children at increased risk of language difficulties in the first years of life. As a synthesis, the findings indicate the high relevance of parent reports, staged combinations with screenings by practitioners, the validity of screenings based on language and language-related skills, the necessity of including home environment variables and factors such as language comprehension and parental concerns that increase predictive validity and—for some of the studies –acceptance by those involved and practicability in public health approaches. The current state of the development of screening procedures warrants their implementation and evaluation in surveillance programs in total population samples.
